# Case of Lodgement and Retention of a Foreign Body in a Bronchus for the Space of 60 Years: Its Expulsion

**Published:** 1846-02

**Authors:** Rodman Bartlett

**Affiliations:** Student of Medicine, Salisbury, Conn.


***Case of Lodgment and Retention of a Foreign Body in a Bronchus for the space of sixty years, with its final expulsion.***

(Communicated by Rodman Bartlett, Student of Medicine, Salisbury, Conn.)—The following case being somewhat unique in its character, may, perhaps, be deemed of sufficient interest to find a place in your valuable journal. It occurred in the practice of my preceptor, Luther Ticknor, M. D., who is ready to vouch for the facts here stated:

Richard Moore, of Salisbury, Conn., now aged sixty-three years, inherited a good constitution, but when three years old, swallowed accidentally a piece of bone, which nearly suffocated him at the time, and which, from that day to the present, has produced a series of phenomena, which could only be accounted for, on the supposition that the bone was lodged in some portion of the bronchial tree. For a long time he was harassed night and day with cough, and severe dyspnœa, attended with a rattling, or sibilant rhoncus, fœtid breath, more or less pain deep in the right side, about opposite the fifth or sixth rib, and midway between the sternum and dorsal vertebræ. These symptoms continued to harass him until nearly the age of puberty, when other symptoms appeared, of a still more alarming nature. At this period, the cough began to be accompanied with expectoration of purulent matter, tinged with blood; attended also with marasmus or atrophy, and other symptoms of confirmed pulmonary disease. At the age of nineteen, he was suddenly attacked with hæmoptysis, which continued, at irregular intervals, for a series of years; during which time his general health and strength were greatly impaired, so that he was incapable of much exertion, being, indeed, confined to the house for more than eight years, About the age of twenty-eight, his general health began to mend; although still troubled with cough. For the next fifteen or twenty years, he was able to perform some labor, though not without considerable inconvenience from the symptoms above mentioned. In January, 1844, for the first time, since the third year of his age, the cough suddenly left him, and did not return for several months, when it again began suddenly to harass him as before. On the 8th of October, 1845, he experienced a remarkably uneasy sensation, of a pricking nature, deep in the right side, which excited violent coughing, and after one or two severe paroxysms, he experienced a sensation as if something had ruptured, or given way. This was instantly followed by the passage of something into the trachea, producing suffocation, which was forcibly ejected upon the floor, succeeded by the expectoration of purulent matter streaked with blood. On examination, the foreign body turned out to be a *bone*, nearly three fourths of an inch in length, one fourth of an inch in breadth, and one twelfth in thickness, oblong and somewhat triangular in form, smooth and convex upon one surface, the other covered with sulci and protuberances. It appeared that the bone which he had originally swallowed, from the traditionary accounts preserved in the family, was a splinter from a rib from which he was sucking the meat, and it seemed highly probable, on examining the piece ejected, that this also was originally a portion of the same bone, as the spongy lamellæ were still visible on one side, while the smooth convex surface of the other was equally manifest. Now, then, are we not authorized in believing that this bone was lodged in one of the rami of the right bronchus for this long period of 60 years, producing the phenomena, which I have briefly described.—*New York Journal of Medicine, and the Collateral Sciences.*

